# The CBL and CIPK Gene Family in Grapevine (*Vitis vinifera*): Genome-Wide Analysis and Expression Profiles in Response to Various Abiotic Stresses

**DOI:** 10.3389/fpls.2017.00978

**Published:** 2017-06-09

**Authors:** Yue Xi, Jinyi Liu, Chao Dong, Zong-Ming (Max) Cheng

**Affiliations:** ^1^Fruit Crop Systems Biology Laboratory, College of Horticulture, Nanjing Agricultural UniversityNanjing, China; ^2^Department of Plant Sciences, University of TennesseeKnoxville, TN, United States

**Keywords:** calcineurin B-like proteins, CBL-interacting protein kinases, gene family, gene expression, grapevine

## Abstract

Calcium plays a central role in regulating signal transduction pathways. Calcineurin B-like proteins (CBLs), which harbor a crucial region consisting of EF hands that capture Ca^2+^, interact in a specific manner with CBL-interacting protein kinases (CIPKs). This two gene families or their interacting-complex widely respond to various environment stimuli and development processes. The genome-wide annotation and specific expression patterns of CBLs and CIPKs, however, in grapevine remain unclear. In the present study, eight *CBL* and 20 *CIPK* genes were identified in grapevine genome, and divided into four and five subfamilies, respectively, based on phylogenetic analysis, and validated by gene structure and the distribution of conserved protein motifs. Four (50%) out of eight *VvCBLs* and eight (40%) out of 20 *VvCIPKs* were found to be derived from tandem duplication, and five (25%) out of 20 *VvCIPKs* were derived from segmental duplication, indicating that the expansion of grapevine CBL and CIPK gene families were mainly contributed by gene duplication, and all duplication events between *VvCIPK* genes only detected in intron poor clade. Estimating of synonymous and non-synonymous substitution rates of both gene families suggested that *VvCBL* genes seems more conserved than *VvCIPK* genes, and were derived by positive selection pressure, whereas *VvCIPK* genes were mainly derived by purifying selection pressure. Expressional analyses of *VvCBL* and *VvCIPK* genes based on microarray and qRT-PCR data performed diverse expression patterns of *VvCBLs* and *VvCIPKs* in response to both various abiotic stimuli and at different development stages. Furthermore, the co-expression analysis of grapevine *CBLs* and *CIPKs* suggested that CBL-CIPK complex seems to be more responsive to abiotic stimuli than during different development stages. *VvCBLs* may play an important and special role in regulating low temperature stress. The protein interaction analysis suggested divergent mechanisms might exist between Arabidopsis and grapevine. Our results will facilitate the future functional characterization of individual *VvCBLs* and *VvCIPKs*.

## Introduction

Calcium serves as a ubiquitous second messenger and plays a critical role in plant against various abiotic stresses. Calcium signals are firstly perceived by Ca^2+^ sensors, such as calmodulin-like proteins (CMLs), calmodulins (CaMs), calcium-dependent protein kinases (CDPKs), and the plant-specific calcineurin B-like proteins (CBLs) (Kudla et al., 2010), and then subsequently relayed into downstream responses in specific manners like interacting with downstream proteins and phosphorylation cascades. Two types of Ca^2+^ sensor proteins, including sensor relays and sensor responders, were classified based on their structural features. Sensors relays include CMLs and CBLs, which do not have kinase activity. They can specifically target downstream proteins to transfer the perceived calcium signals in response to various environmental stimuli and development processes. Sensor responder proteins, like CaMs and CDPKs, have all the functions of Ca^2+^ sensor relay proteins as well as the kinase activity. CBLs contain a crucial structural component, which consists of four common helix-loop-helix structure motifs (EF hands) as a calcium-binding site to capture Ca^2+^, and is responsible to interact in a specific manner with CBL-interacting protein kinases (CIPKs), which also known as sucrose non-fermenting 1 (SNF1) related kinase 3 (SnRK3) (Xiang et al., 2007). Importantly, the linkers region between each EF hand motifs are absolutely conserved in all CBL proteins. Generally, there are 22 amino acids between EF1 and EF2, 25 amino acids between EF2 and EF3, and 32 amino acids between EF3 and EF4. In contrast, the EF hands, which consist of a loop of 12 amino acids, are less conserved and carried the variations that contribute to the function diversity or the interacting property of CBL (Zhang et al., 2008). Additionally, several CBL proteins possess an N-myristoylation site domain which is required to function in plant salt tolerance (Ishitani et al., 2000). Conserved serine residues in PFPF motif of CBL proteins are also required for phosphorylation of CBLs by CIPKs (Du et al., 2011). CIPK proteins are made up of an N-terminal serine/threonine protein kinase domain and a self-inhibitory C-terminal regulatory domain (Albrecht et al., 2001). A conserved NAF/FISL motif, required for interacting with CBLs, is present in the C-terminal regulatory domain of CIPKs, and is responsible for the activation role of CIPKs (Albrecht et al., 2001; Guo et al., 2001). Another protein-phosphatase interaction (PPI) domain present in the C-terminal of a few CIPKs can specifically target several different phosphatase 2C (PP2C) proteins (Ohta et al., 2003).

In previous research, the CBL-CIPK complex has been reported to play a significant role in plant response to abiotic stress and nutrient signaling cascades (Li et al., 2009; Weinl and Kudla, 2009; Yu et al., 2014). Increasing number of CBLs and CIPKs has been demonstrated to play a role in enhancing stress tolerance by regulating the intracellular ion concentration in plants, functional analysis of several CBLs and CIPKs in Arabidopsis has shown that the CBL-CIPK network plays an important role in regulating sodium (Na^+^), potassium (K^+^), and nitrate (NO^3−^) transport across the plasma membrane and tonoplast, as well as in auxin and abscisic acid (ABA) signaling, and a variety of developmental processes (Luan et al., 2009; Weinl and Kudla, 2009). The CBL-CIPK complex was first identified in the salt overly sensitive (SOS) pathway from Arabidopsis. CBL4 (SOS3) was shown to interact with CIPK24 (SOS2) and recruit it to the plasma membrane, where the complex activates the Na^+^/H^+^ antiporter (SOS1) located on the plasma membrane and the vacuolar H^+^-ATPase, resulting in enhanced salt tolerance (Qiu et al., 2002). The CBL4-CIPK6 complex was shown to modulate the activity of the plasma membrane K^+^ channel AKT2 in plant cells by mediating the translocation of AKT2 to the plasma membrane and enhancesAKT2 activity in *oocytes* (Held et al., 2011). CIPK6 and CIPK16 from Arabidopsis have been demonstrated to be able to interact with AKT1 in *Xenopus oocytes* (Lee et al., 2007). The transgenic cotton plants overexpressing GhCIPK6 conferred tolerance to a variety of abiotic stresses (He et al., 2013). Overexpression of SICIPK24 (SISOS2) increases salt tolerance in tomato (Huertas et al., 2012). Arabidopsis CIPK6 is also required for growth and development in plants. In Arabidopsis, a lesion in CIPK6 reduced basipetal auxin transport and plants exhibited fused cotyledons, swollen hypocotyls, and compromised lateral root formation (Tripathi et al., 2009). AtCIPK8 was reported to regulate the low-affinity phase of the nitrate primary response (Hu et al., 2009). The expression of AtCIPK3 increased tolerance to ABA and low temperature, high salt, wounding, and drought stress in Arabidopsis and shown to modulate abscisic acid and low temperature signal transduction (Kim et al., 2003). Arabidopsis CIPK26 interacts with an early establishment RING-type E3 ligase and Keep on Going (KEG) components of the ABA signaling network (Lyzenga et al., 2013). OsCIPK31 is involved in germination and seedling growth of rice plants subjected to abiotic stress conditions (Piao et al., 2010). Besides, AtCBL10 was reported to interact with AtCIPK24 in response to salt stress (Kim et al., 2007). CBL10 was also shown to compete with CIPK23 for binding to AKT1, thus negatively modulating the activity of AKT, but the direct interaction of CBL10 with AKT1 in Arabidopsis indicates that CBLs can affect downstream components independent of CIPK protein (Ren et al., 2013). CBL1 or CBL9 can interact with CIPK23 to promote potassium uptake under low K^+^ condition by phosphorylating and activating the K^+^ channel AKT1in Arabidopsis and rice roots (Xu et al., 2006; Cheong et al., 2007; Li et al., 2014). CBL1 and CBL9, however, may also interact with AKT1 independent of CIPK2 (Ye et al., 2013). Collectively, the above mentioned studies demonstrate that CBLs and CIPKs play important roles in response to abiotic stress and development processes in plants.

Grapevine (*Vitis vinifera*) is one of the most widely grown and most economically valuable fruit crops in the world. Abiotic stresses, such as drought and salt stresses in particular, threatened the growth of grapevine world widely, thereby affect fruit yield and quality (Ma et al., 2015). CBLs and CIPKs as the key components of plant perceiving and relaying calcium signals play crucial role in plant response to abiotic stresses. In grapevine, CBL-CIPK network has been indicated to activate shaker inward K^+^ channel which is very important for fruit development and strongly up-regulated by drought stress (Cuellar et al., 2010). However, genome-wide analysis and the specific regulatory mechanism and function diversity of CBLs and CIPKs remain uncovered. In the present study, total of 8 CBLs and 20 CIPK family members were identified and their phylogenetic relationship, gene structure, protein motifs, promoter, gene duplication, and divergence were analyzed. Furthermore, the expression profiles of *VvCBLs* and *VvCIPKs* in response to various abiotic stresses and in different tissues and their developmental stages was characterized using reverse transcription-quantitative polymerase chain reaction (RT-qPCR) and a publicly available microarray data, respectively. In addition, co-expression analysis and protein interaction prediction provide an overall functional conservation and divergence of CBL-CIPK complex in grapevine. Our results will facilitate the future functional characterization of individual *VvCBLs* and *VvCIPKs* in responses to stresses and developmental signals.

## Materials and methods

### Genome-wide identification of *CBL* and *CIPK* genes in grapevine

The most recent version (V2.1) of a 12X assembly of the grapevine genome (*V. vinifera*) was downloaded from CRIBI (http://genomes.cribi.unipd.it/) for use in identifying grapevine CBL and CIPK proteins. Additionally, 10 CBL and 33 CIPK genes from rice (*Oryza sativa*) were downloaded from the rice genome database (http://rice.plantbiology.msu.edu//), and 10 CBL and 26 CIPK genes from Arabidopsis were downloaded from TAIR (https://www.arabidopsis.org/index.jsp) database. Grapevine CBL and CIPK genes were identified using the following two steps: (i) the well-characterized Arabidopsis 10 CBLs and 26 CIPKs were used as queries against the grapevine genome using BLASTP with e-values <1E-5. (ii) The identified grapevine CBL and CIPK genes were further confirmed by constructing a phylogenetic tree using the Neighbor-Joining (NJ) method of identified grapevine CBLs and CIPKs and the CBLs and CIPKs from Arabidopsis and rice. Only grapevine genes with an E-value <10^−4^, and an identy >55%, and that located in one the clades of derived from Arabidopsis and rice *CBL* and *CIPK* genes were regarded as *VvCBL* and *VvCIPK* genes, respectively. The genes considered as grapevine CBLs and CIPKs were then named in accordance with the gene nomenclature rules established by the grapevine scientific community (Grimplet et al., 2014). *VvCBLs* and *VvCIPKs* names were assigned based on their ortholog genes in Arabidopsis identified using bootstrap replicates of the Maximum Likelihood phylogenetic tree with values higher than 70. Only one-to-one orthologs were considered when allocating an Arabidopsis-like name to the *Vitis* gene. Otherwise, the names of the *VvCBL* and *VvCIPK* were assigned a number higher than the highest number used in the naming of Arabidopsis *CBL* and *CIPK* genes. The molecular weight (MW) and isoelectric point (pI) of each protein sequence were calculated using ExPASY (http://web.expasy.org/compute_pi/).

### Phylogenetic analysis

The protein sequence of all of the identified CBL and CIPK family members from grapevine, Arabidopsis, rice and poplar (*Populus* sp.) were aligned using Muscle. Phylogenetic trees were constructed using the Neighbor-Joining (NJ) method with the MEGA6.0 software program with 1,000 bootstrap replicates and the Poisson model. The classification of subfamilies of grapevine CBL and CIPK genes was consistent with the previous subfamilies reported in Arabidopsis and poplar (Yu et al., 2007; Zhang et al., 2008).

### Gene structure and conserved motifs for VvCBLs and VvCIPKs

The coding DNA sequences (CDS) and genomic sequences of grapevine CBLs and CIPKs were retrieved from the CRIBI genomic database for grapevine. Gene structures of *VvCBLs* and *VvCIPKs* was analyzed using the Gene Structure Display Server (GSDS 2.0, http://gsds.cbi.pku.edu.cn/). Prediction of motifs were generated using the Multiple Em for Motif Elicitaton (MEME) program (http://meme-suite.org/tools/meme), with an optimum width of motifs ranging from 6 to 50, the maximum number of motifs set at 18, and default values for other parameters.

### Chromosomal location and gene duplication of *VvCBL* and *VvCIPK* genes

The chromosomal locations of grapevine *CBL* and *CIPK* genes were verified from the CRIBI database, and chromosomal images were drawn using MapInspect software. Tandem duplicated genes were defined as an array of two or more genes located on the same chromosome and found within a 100 kb genomic window (Zhu et al., 2016). Segmental duplication genes were identified as genes located on duplicated chromosomal blocks, and were determined using MCScanX software (http://chibba.pgml.uga.edu/mcscan2/) to detect gene duplication events using an E-value 10^−5^.

### Evolutionary analysis

To better understand the patterns of macroevolution, the non-synonymous substitutions (Ka) and synonymous substitutions (Ks) rates and diverse time of *CBLs* and *CIPKs* from grapevine, rice, Arabidopsis, and poplar were extensively estimated. Firstly, the nucleotide coding sequences (CDSs) of duplicated *VvCBL* genes and *VvCIPK* genes were aligned using ClustalW 2.0 (Larkin et al., 2007), and then non-synonymous substitutions, synonymous substitutions, and the ratio between them (Ka/Ks) were calculated using MEGA 5.0 (Tamura et al., 2011). Secondly, the Ks value was used to calculate the time (Mya, million years ago) of duplication as T = Ks/2λ (λ = 6.5 × 10^−9^ for grape) (Cao et al., 2014). The collinearity relationship between grapevine and other species including rice, poplar, and Arabidopsis were retrieved from Plant Genome Duplication Database (PGDD, http://chibba.agtec.uga.edu/duplication/).

### Promoter analysis

The 2,000 bp upstream sequences of coding region of *VvCBL* and *VvCIPK* genes were downloaded from CRIBI (http://genomes.cribi.unipd.it/). The cis-regulatory elements were identified using PlantCARE (http://bioinformatics.psb.ugent.be/webtools/plantcare/html/) software (Wang et al., 2015).

### Plant material and experimental treatments

“Pinot Noir” grape (*V. vinifera*, the sequenced genotype PN40024) plantlets were grown on MS medium in a tissue culture room with a photoperiod of 16 h light/8 h dark and a temperature of 23°C for 6 weeks.

Salinity and drought stress treatments were administered by irrigating the plants with either 200 mM NaCl or 10% polyethylene glyco 6000 (PEG), respectively. For the nutrient stress treatment (Xu et al., 2006), plants were irrigated with a modified 1/2 MS solution containing only 100 μM K^+^ (Xu et al., 2006). Low temperature and heat stress treatment were applied by putting the plants at 4°C (Drug storage box, HYC-360, Haier) and 42°C (Intelligent artificial climate box, RXZ-380C, Jiangnan Instrument Factory, Ningbo) with a photoperiod of 16 h light/8 h dark, respectively. Leaves in all the treatments were harvested at 0, 6, 12, and 24 h post treatment application. The collected leaves were immediately frozen in liquid nitrogen and stored at −70°C until further analysis. Three biological replicates (three independent plants) were used in all the stress treatments and the corresponding controls.

### qRT-PCR and microarray data

Total RNA was extracted from the sampled leaves using a previously described protocol (Gonzalez-Mendoza et al., 2008). The concentration and purity of RNA were determined by measuring the optical density (OD) ratio at 260 and 280 nm using a One Drop™ OD-1000 spectrophotometer (Thermo Fisher Scientific, USA). RNA integrity was evaluated by electrophoresis on a 1% agarose gel stained with ethidium bromide. A high capacity Prime Script™ RT reagent Kit with gDNA Eraser (Takara, Japan) was used to eliminate traces of genomic DNA and to synthesize first strand cDNA from the template RNA according to the manufacturer's instructions.

The specific expression of *VvCBLs* and *VvCIPKs* was examined by RT-qPCR using the SYBR Green method on a Bio-Rad CFX 96 PCR real-time thermo cycler. The oligo nucleotide primers for amplifying specific *VvCBLs* and *VvCIPKs* were designed using Primer Premier 5 (http://www.premierbiosoft.com/primerdesign/) software, and were based on the 3′-untranslated region (UTR) and the 3′ terminal sequence of the coding region (Wang G. et al., 2014). The housekeeping gene (actin-101-like, VIT_012s0178g00200) was used for normalization of the data (Wang M. et al., 2014; Liu et al., 2016). All primer sequences are listed in Table S5. The total volume of each RT-qPCR reaction was 20 μL which included 2 μL cDNA from each sample as a template, 10 μL SYBR Premix Ex TaqTM (Tli RNase Plus, Takara, Japan), 0.8 μL of each primer, and 6.4 μL ddH_2_O. The PCR conditions consisted of denaturation step for 30 s at 95°C, followed by 40 cycles of 5 s at 95°C and 60°C for 20 s at the end. The melting curve from 65°C to 95°C for 15 s for each amplification was conducted immediately after the completion of the qRT-PCR to verify the specificity of each amplification. Three replicates were run for each treatment sample and the corresponding controls. Relative expression values (fold change) were calculated using the 2^−ΔΔCT^ method (ΔCT = CT target – CT reference; ΔΔCT = (CT target-CT reference) treatment -(CT target-CT reference)control) (Livak and Schmittgen, 2001). The relative expression of each of the examined genes was represented as values relative to the untreated controls. Mean values ± standard deviation (*SD*) were calculated from the three biological replicates.

High-throughput microarray data from published research (Fasoli et al., 2012) were downloaded from the Gene Expression Omnibus (GEO, https://www.ncbi.nlm.nih.gov/geo/) database at the National Center for Biotechnology Information (NCBI) to characterize the general spatial and temporal expression patterns of *VvCBL* and *VvCIPK* genes during development. In the analysis of the microarray data, 54 samples from green and woody tissues and organs of grapevine at different developmental stages (GSE36128) were examined, and the log10 value of gene expression was used. The derived heatmaps were drawn using Multi experiment viewer 4.9 (MeV, http://www.tm4.org/mev.html) software.

### Co-expression network and protein interaction of CBLs and CIPKs

The Pearson correlation coefficient (PCC) value was calculated between each pair of *VvCBLs* and *VvCIPK* using gene expression values from the high-throughput transcriptome data and qRT-PCR data using Statistical Product and Service Solutions (SPSS v20.0) software. Co-expressed gene pairs were filtered with a PCC cut-off of 0.7 (Liu et al., 2016).

For the protein interaction networks, high confidence, experimental data of interactive CBL and CIPK proteins in Arabidopsis were constructed by using STRING (http://string-db.org/) using an option value > 0.7. The homolog proteins of the determined interactive Arabidopsis proteins were identified in grapevine by reciprocal best BLASTP analysis.

### Statistical analysis

All results are presented as means ±*SD* of three biological replicates where each biological replicate consisted of three technical replicates. Significant statistical differences between treatments were determined by a Student's test (*P* < 0.05) using SPSS.

## Results

### Identification of *CBL* and *CIPK* genes in grapevine

Based on the BLAST query and subsequent phylogenetic tree construction (Figure S1, Table S1), a total of eight non-redundant CBLs and 20 CIPKs were identified from the latest version (V2.1) of grapevine genome annotation, respectively (Table 1). Among them, the sequences of *VvCBL12* was manually corrected due to its ambiguous annotation from V2.1 genome annotation (see the notes of Table 1). The grapevine *CBLs* and *CIPKs* were then named according to the nomenclature rules recently established by the grapevine scientific community (Grimplet et al., 2014). The detailed information of the identified *VvCBLs* and *VvCIPKs* are listed in Table 1.

**Table 1:** Features of *CBL* and *CIPK* genes in grapevine.

**Gene ID**	**Gene name**	**Chr**	**locus**		**chain**	**Protein length**	**PI**	**MW**
VIT_200s1569g00020^f^	VvCBL12	Un	39588889	39592698	−	226	4.71	25.89
VIT_219s0015g01070	VvCBL11	19	9147637	9157623	+	226	4.79	26.01
VIT_204s0008g00950	VvCBL10a	4	833260	838903	+	258	5.02	29.81
VIT_204s0008g00960	VvCBL10b	4	841037	848429	+	251	4.88	28.99
VIT_202s0236g00140	VvCBL13	2	5573432	5598446	+	213	4.68	24.47
VIT_202s0025g01640	VvCBL8	2	1578845	1583355	–	219	4.71	25.24
VIT_216s0098g01870	VvCBL4	16	21954020	21960817	+	213	4.72	24.52
VIT_202s0025g01630	VvCBL5	2	1575403	1577828	−	207	4.70	23.65
VIT_206s0004g07830	VvCIPK38	6	8605061	8608169	+	448	9.27	50.67
VIT_213s0067g02480	VvCIPK37	13	1344039	1347821	+	464	9.14	52.65
VIT_208s0058g01040	VvCIPK34	8	10381694	10385778	+	462	8.84	51.99
VIT_216s0022g00350	VvCIPK35	16	11280909	11281763	+	177	9.24	20.97
VIT_210s0003g01410	VvCIPK36	10	2789114	2792146	+	467	8.41	52.73
VIT_209s0070g00160	VvCIPK30	9	13197151	13198689	+	398	8.73	44.64
VIT_211s0016g00200	VvCIPK31	11	230595	232286	−	426	9.10	47.74
VIT_204s0008g05770	VvCIPK32	4	5291827	5293589	−	447	8.99	50.55
VIT_209s0070g00140	VvCIPK33	9	13115563	13117457	+	461	8.72	52.25
VIT_210s0003g01420	VvCIPK12	10	2803127	2807265	−	502	6.88	56.35
VIT_205s0020g04570	VvCIPK29	5	6388841	6390574	+	420	8.93	46.51
VIT_206s0004g07870	VvCIPK28	6	8640468	8642137	−	436	8.59	48.71
VIT_208s0058g01090	VvCIPK27	8	10443101	10444884	−	459	6.66	52.40
VIT_218s0001g07980	VvCIPK41	18	6442668	6452885	−	447	6.72	50.62
VIT_211s0016g05420	VvCIPK24	11	4753080	4787687	−	416	8.53	47.22
VIT_210s0003g04020	VvCIPK39	10	6827796	6838354	+	431	8.94	48.42
VIT_215s0048g02740	VvCIPK9	15	16877139	16882647	−	441	8.50	49.93
VIT_206s0009g01840	VvCIPK3	6	13941393	13952395	+	439	6.88	50.09
VIT_211s0052g01700	VvCIPK21	11	19454809	19458498	−	472	5.65	53.21
VIT_205s0020g00830	VvCIPK40	5	2672344	2676586	−	391	6.06	44.05

f *This gene was re-annotated from the ORCAE platform by the authors (http://bioinformatics.psb.ugent.be/orcae/overview/Vitvi)*.

Furthermore, the multiple amino acids sequence alignments of VvCBLs along with AtCBLs were shown in Figure S2A, indicating that general structures of VvCBLs protein are highly conserved, with all VvCBLs having four EF hands similar to those of EF-hand motifs in AtCBLs. All CBL proteins have invariant spacing between each EF-hand motif (Figure S2A). A total of 23 amino acids lie between EF1 and EF2, 25 amino acids between EF2 and EF3, and 32 amino acids betweenEF3 and EF4. Notably, a recently identified PFPF motif, which is used for phosphorylation of CBLs and CIPKs (Kanwar et al., 2014), was also identified, and which is highly conserved in all eight *VvCBLs* (Figure S2A). In addition, three VvCBLs, including VvCBL13, VvCBL4, and VvCBL5, were found to harbor a myristoylation site in the N-terminal sequence (Figure S2A).

Similarly, the multiple sequence alignment of VvCIPK proteins shown that all the VvCIPKs contain an N-terminal catalytic kinase domain and a C-terminal regulatory domain which were necessary and very important for protein-protein interaction, except VvCIPK35, which had the high homology with other Arabidopsis CIPKs but seems to be missing both the C-terminal regulatory domain and the NAF domain. In addition, the other VvCIPKs also possess an activation domain in the N-terminal sequence as well as a NAF domain in the C-terminal sequence simultaneously.

### Phylogenetic analysis of *VvCBL* and *VvCIPK* genes

**Figure 1:**
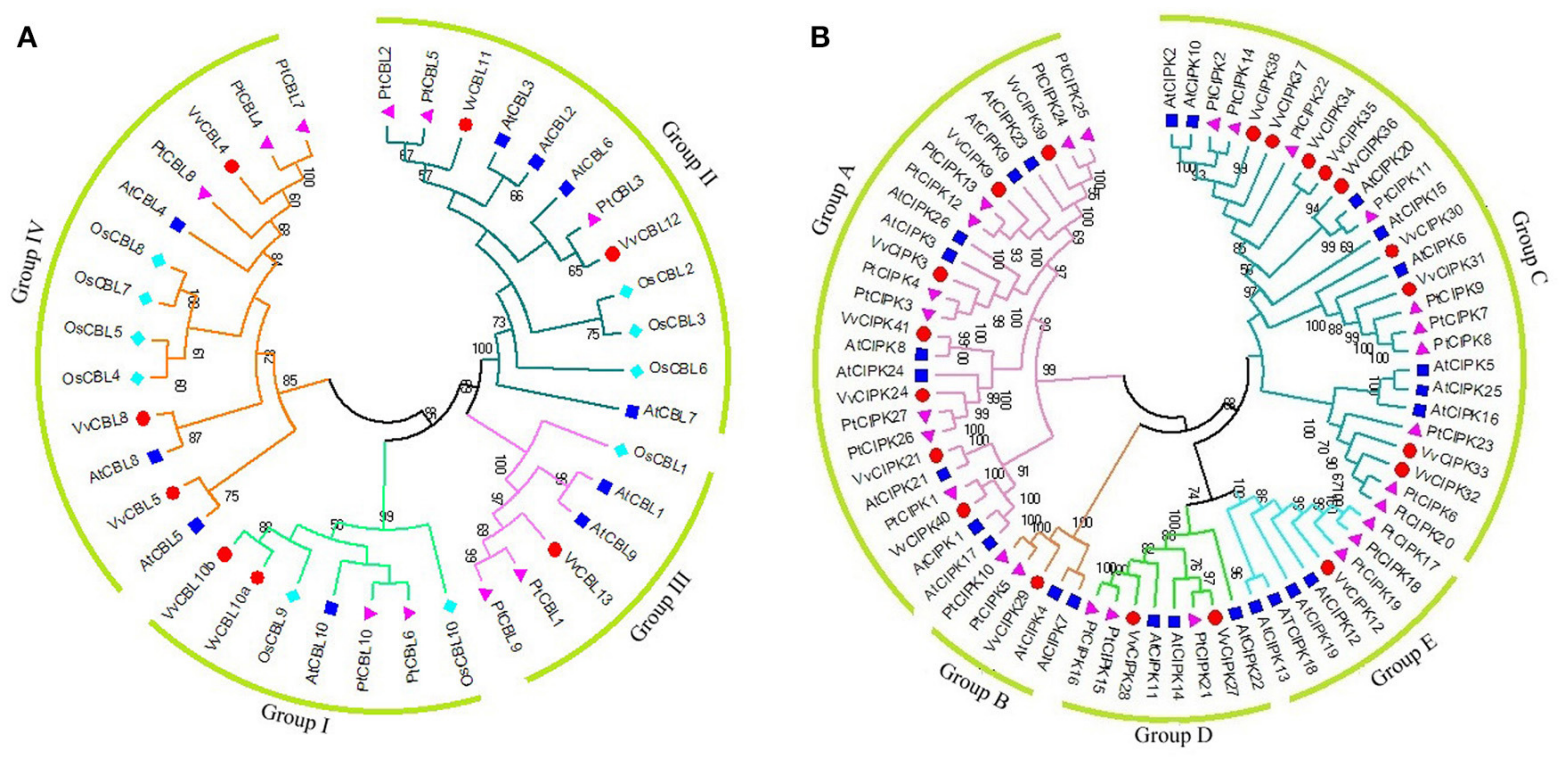
The phylogenetic analysis of CBL **(A)** and CIPK **(B)** gene families across grapevine, Arabidopsis, rice, and poplar. Full length protein sequences of CBLs and CIPKs were used to construct Neighbor-Joining (NJ) trees by using MEGA6.0 software with bootstrap value 1,000. Subfamilies are highlighted with different colors. The grapevine CBL and CIPK genes were marked by red dots. The poplar *CBL* and *CIPK* genes were marked by purple Triangle. The Arabidopsis *CBL* and *CIPK* genes were marked by blue square. The rice *CBL* and *CIPK* genes were marked by cyan rhombus.

In order to investigate the evolutionary relationships between grapevine CBL and CIPK proteins and other species, a Neighbor-Joining phylogenetic tree was constructed using the full amino acid sequences of CBL and CIPK family proteins from grapevine, Arabidopsis, rice, and poplar (*Populus* sp.). The CBLs and CIPKs were clustered into four (Figure 1A) and five (Figure 1B) subfamilies, respectively. As shown in Figure 1A, VvCBL10a and VvCBL10b were clustered in group I and were identified as orthologous gene pairs with OsCBL9. However, VvCBL11 and VvCBL12 formed as group II, and VvCBL13 formed as Group III, which seemed to be more close to poplar CBLs. In group IV, VvCBL5 and VvCBL8 were homologous to AtCBL5 and AtCBL8, respectively, while the VvCBL4 seems to be more close to popular CBLs. The variant phylogenetic relationships of VvCBLs indicated that the *CBL* genes have different evolutionary rate during grapevine evolution.

In regards to the 20 VvCIPKs, group A included VvCIPK3, -9, -21, -24, -39, -40, -41, group B included only VvCIPK29, group C included VvCIPK30, -31,-32, -33, -34, -35, -36, -37, -38, group D included VvCIPK27 and VvCIPK28, and group E included only VvCIPK12. Careful examination of the phylogenetic relationships of CIPKs between grapevine and other two species (Arabidopsis and poplar) showed that phylogenetic relationships of VvCIPKs were more close to poplar comparing that to Arabidopsis, indicating that the evolutionary rate of grapevine CIPK gene family is faster than what they supposed to be.

Furthermore, Closely-related orthologous pairs of CBLs and CIPKs were identified between grapevine and Arabidopsis based on high bootstrap value. Such as VvCBL8 and AtCBL8, VvCBL10a, -10b and ATCBL10, VvCBL4 and AtCBL4, VvCBL5 and AtCBL5, VvCIPK3 and AtCIPK3, VvCIPK9 and AtCIPK9, VvCIPK12 and AtCIPK12, VvCIPK21 and AtCIPK21, VvCIPK24 and AtCIPK24. All bootstrap values are higher than 70 and ranged from 70 to 100, suggesting that an ancestral set of CIPK and CBL genes existed prior to the divergence of grapevine and Arabidopsis.

### Gene structure and conserved motifs of grapevine CBLs and CIPKs

**Figure 2:**
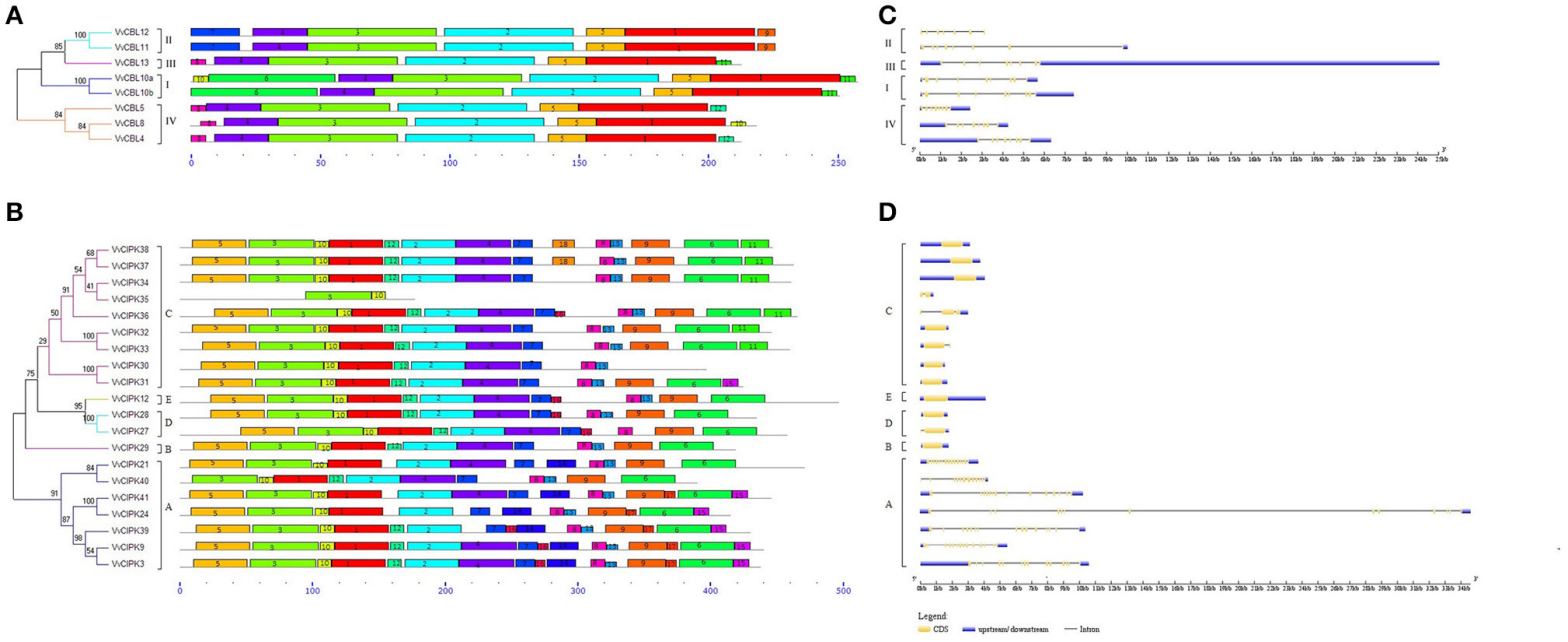
Conserved motifs of grapevine CBL **(A)** and CIPK **(B)** proteins and gene structures of grapevine *CBL* **(C)** and *CIPK* **(D)** genes. The conserved motifs of VvCBL and VvCIPK proteins were performed using MEME program and arranged corresponding to the phylogenetic tree. Different motifs are highlighted with different color boxes and numbers. The length of boxes corresponded to motif length. The gene structures were drawn using the GSDS program. The yellow boxes represent exons, the lines represent introns and the blue boxes represent upstream/downstream UTRs. Subgroup B, C, D, and E of grapevine CIPK family constituted an obviously intron poor clade.

Intron/exon organization and conserved motifs were analyzed in order to further investigate the structural features of grapevine *CBLs* and *CIPKs*. As illustrated in Figure 2C, *VvCBLs* genes in group I have the greatest number of introns with eight, genes in group III and group IV have seven introns, as does *VvCBL11* in group II. *VvCBL12* in group II has six introns. *VvCIPK* family members clustered into an intron-rich clade (> 8 introns per gene) and an intron-poor clade (<3 introns per gene) (Chen et al., 2011; Ye et al., 2013). The intron-rich *VvCIPK* genes clustered in subgroup A, while the intron-poor genes are present in all four of the other subgroups B, C, D, and E (Figure 2D). Within the intron-poor clades, only *VvCIPK36* in subgroup C has two introns, while *VvCIPK35* and *VvCIPK33* in subgroup C, and *VvCIPK27* in subgroup D, have one intron. The remaining CIPK genes have no intron.

Furthermore, the conserved motifs of VvCBLs (Figure 2A) and VvCIPKs (Figure 2B) were determined by using MEME software. Twelve conserved motifs were detected in VvCBL proteins and the detailed information about each motif is presented in Figure S3A. All of the grapevine CBL proteins contain motif 1 to motif 5, which were also annotated as the four EF-hand domain and PFPF motif, respectively. However, motif 6 is only present in subgroup I, and both of motif 7 and 9 are only present in subgroup II, suggesting that those motifs might play important role specific to corresponding subgroups.

A total of 18 motifs were identified in VvCIPK proteins (Figure 2B) and amino acid sequences of each motif are presented in Figure S3B. Motif 8 is the functional NAF domain of CIPK proteins and is widely distributed in all of the VvCIPK proteins, except VvCIPK35, which only has motifs 3 and 10 within the N-terminal catalytic kinase domain. VvCIPK40 appears to have lost motif 5, which also annotated as an ATP-binding domain. Intron-rich VvCIPK proteins possess motif 17, except VvCIPK21 and VvCIPK40 in group A. CIPK proteins in group C all possess motif 11, except VvCIPK30, VvCIPK31, and VvCIPK35.

### Gene duplication and divergence of *VvCBL* and *VvCIPK* genes

**Figure 3:**
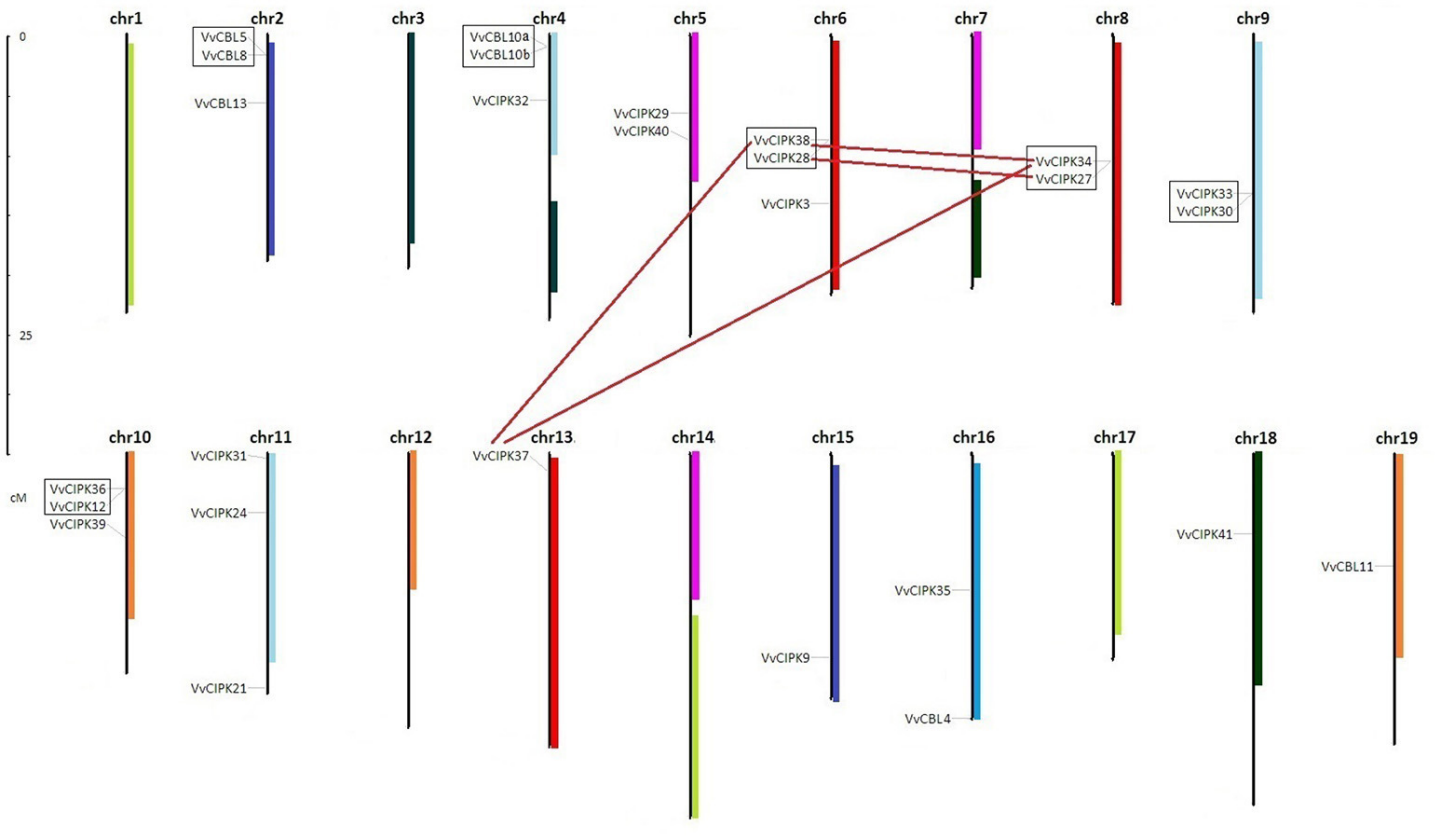
Chromosomal locations of grapevine *CBL* and *CIPK* genes. The 7 *CBL* and 20 *CIPK* genes of grapevine were mapped to 13 chromosomes in which the hidden *VvCBL12* were mapped to chromosomes Un. The segmental duplicated gene pairs were connected by lines. The tandem duplicated gene pairs were marked with boxes.

To understand the functional divergence of grapevine *CBL* and *CIPK* genes, the chromosome distribution and gene duplication events were surveyed (Figure 3). The locations of each *VvCBL* and *VvCIPK* gene were given a diagrammatic representation based on the latest version (V2.1) of grapevine genome annotation. The results showed that the distribution of *VvCBL* and *VvCIPK* genes in chromosomes is not even. 7 *VvCBL* genes are distributed on 4 grapevine chromosomes (*VvCBL12* which locates to chromosome Unknown is not shown). Among them, three *VvCBL* genes is situated on chromosome 2, chromosome 4 has two *VvCBL* genes, and other two located each in chromosomes 16 and 19, respectively (Figure 3, Table 1). The 20 *VvCIPK* genes were mapped to 11 chromosomes (Figure 3). Chromosomes 4, 13, 15, 16, 18 have one *VvCIPK* gene, chromosomes 5, 8, and 9 have two *VvCIPK* genes, and chromosomes 6, 10, and 11 have three *VvCIPKs*. No *VvCIPK* genes are located on grapevine chromosomes 1, 3, 7, 12, 14, and 17 (Figure 3, Table 1). Furthermore, the gene duplication events were analyzed by using MCScanX program and mapped into chromosome location. As shown in Figure 3, two tandem duplication events were detected between 4 *VvCBL* genes, accounting for 50% of *VvCBL* gene family. The gene pairs include *VvCBL5/VvCBL8* (which belong to subgroup IV) and *VvCBL10a/VvCBL10b* (which belong to subgroup I). In contrast, no segmental duplications of *VvCBL* genes were detected. Four tandem duplication events were detected between 8 out of 20 (40%) grapevine *CIPK* genes including *VvCIPK38/VvCIPK28, VvCIPK34/VvCIPK27, VvCIPK33/VvCIPK30*, and *VvCIPK36/VvCIPK12*. Additionally, four segmental duplication events were detected between 5 out of 20 (25%) *VvCIPK* genes. The gene pairs include *VvCIPK38/VvCIPK34, VvCIPK38/VvCIPK37, VvCIPK37/VvCIPK34* of subgroup C, and *VvCIPK28/VvCIPK27* of subgroup D, and among them, 4 *VvCIPK* genes including *VvCIPK38/VvCIPK28, VvCIPK34/VvCIPK27* have already shown to be derived from tandem duplication events. Interestingly, all gene duplication events of *VvCIPKs* were only detected in intron-poor clade (Figure 2).

**Figure 4:**
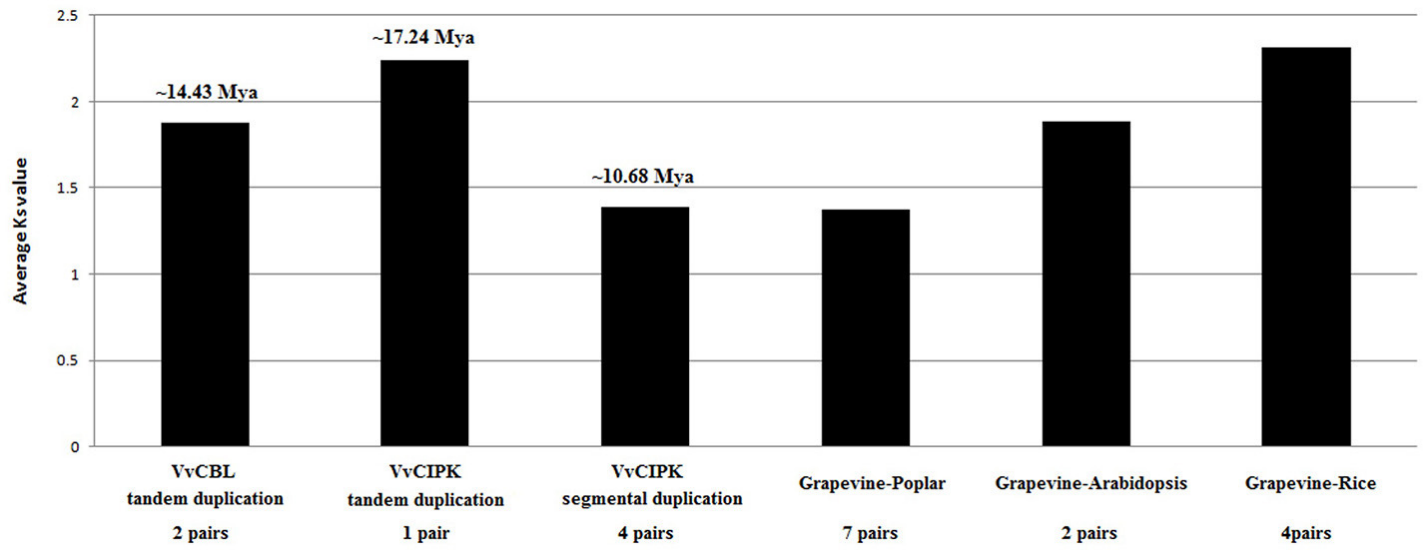
Duplication and divergence based on synonymous substitution rate (Ks) estimated using paralogous and orthologous *VvCBL* and *VvCIPK* gene pairs.

In addition, to better understand the duplication and functional divergence of *VvCBL* and *VvCIPK* genes during their evolution course, the Ka, Ks, and Ka/Ks ratio were calculated. As shown in Figure 4, Table S2, the Ka/Ks ratio of two *VvCBL* tandem duplicated gene pairs were 1.02 and 1.26 with an average of 1.14, and these tandem duplication events of *VvCBL* genes therefore were likely happened around 11 to 17 averaged about 14 Mya (Figure 4, Table S2). The Ka/Ks ratio of one of *VvCIPK* tandem duplicated gene pair was 0.26 and which of four *VvCIPK* segmental duplicated gene pairs were ranged from 0.11 to 0.14 with an average of 0.26. Tandem duplication events of *VvCIPK* genes therefore might happen around 17 Mya, and segmental duplication events of *VvCIPK* genes might happen around 8 to 14 averaged about 11 Mya (Figure 4, Table S2). However, No *VvCBL* orthologous gene pairs with Arabidopsis, rice and poplar were identified. On the contrary, 7 *CIPK* orthologous gene pairs existed between grapevine and poplar with an average Ka/Ks ratio of 0.11, 2 *CIPK* orthologous gene pairs existed between grapevine and Arabidopsis with an average Ka/Ks ratio of 0.06, 4 *CIPK* orthologous gene pairs existed between grapevine and rice with an average Ka/Ks ratio of 0.14 (Table S3). The data indicated that *CIPK* genes among these species were under strong purifying selection.

### Promoter analysis of *VvCBL* and *VvCIPK* genes

To gain more insight into the regulatory mechanism of *VvCBL* and *VvCIPK* genes, The *cis*-acting elements in 2,000 bp upstream sequences of coding region of *VvCBL* and *VvCIPK* genes were surveyed by PlantCARE software. Besides the basic TATA and CAAT boxes and other uncertainty function *cis*-acting, 80 *cis*-acting element were detected, and among them, 65 well-function annotated *cis*-acting were arbitrarily divided into four main kinds of *cis*-acting elements based on their biological functions (Table S4). The first kind was light responsive related elements such as AE-box, rbcS-CMA7a, G-box, SP1, and Box I (Table S4). The Second type was hormone responsive elements, such as GARE-motif, CGTCA-motif, TGA-element, TCA-element, and TATC-box (Table S4). The third type was environment stress related elements, as shown in Table S4, all *VvCBL* and *VvCIPK* genes were found to contain HSE, a *cis*-acting element involved in heat response except *VvCBL12* and *VvCIPK31*. *VvCBL4, VvCBL13, VvCBL5, VvCIPK38, VvCIPK37, VvCIPK34, VvCIPK30, VvCIPK31, VvCIPK32, VvCIPK33, VvCIPK12, VvCIPK29, VvCIPK27, VvCIPK9, VvCIPK3, VvCIPK40* contained MBS, which is MYB binding site involved in drought-deducibility. *VvCBL5, VvCIPK32, VvCIPK12, VvCIPK41, VvCIPK40* contained LTR, which is a *cis*-acting element involved in low temperature responsiveness. WUN-motif, Box-W1, ARE, GC-motif, TC-rich repeats were also detected in *VvCBL* and *VvCIPK* genes. The last kind was plant development *cis*-acting elements, such as Skn-1_motif, which is a *cis*-acting regulatory element required for endosperm expression, it was detected in all *VvCBL* and *VvCIPK* genes except *VvCIPK21* (Table S4), and As-2-box, which involved in both shoot specific expression and light responsive (Table S4), and it was detected in *VvCBL13, VvCIPK34, VvCIPK41*, and *VvCIPK21*.

### The expression profiles of *VvCBLs* and *VvCIPKs* in response to various abiotic stresses and in different developmental stages of grapevine

**Figure 5:**
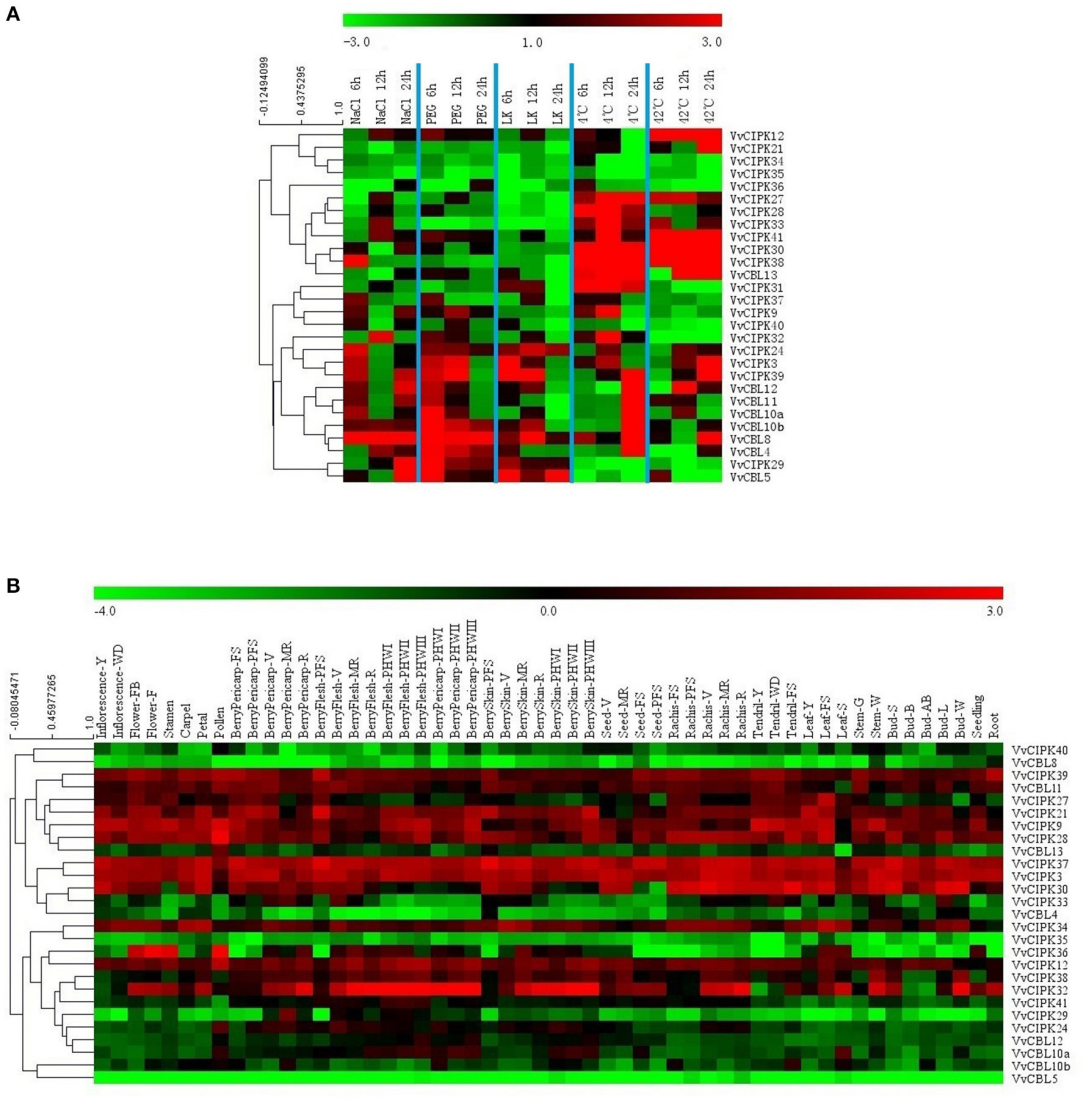
Expression profiles of VvCBL and VvCIPK genes in response to salt, PEG, low potassium, cold, and heat **(A)** and in 54 different tissues and development stages based on a high-throughput transcriptome data **(B)** (Fasoli et al., 2012). Genes were hierarchically clustered based on the average Pearsion's distance. Relative expression pattern of **(A)** were determined by qRT-PCR. Fluorescence intensities values of **(B)** were log_2_-based.

**Figure 6:**
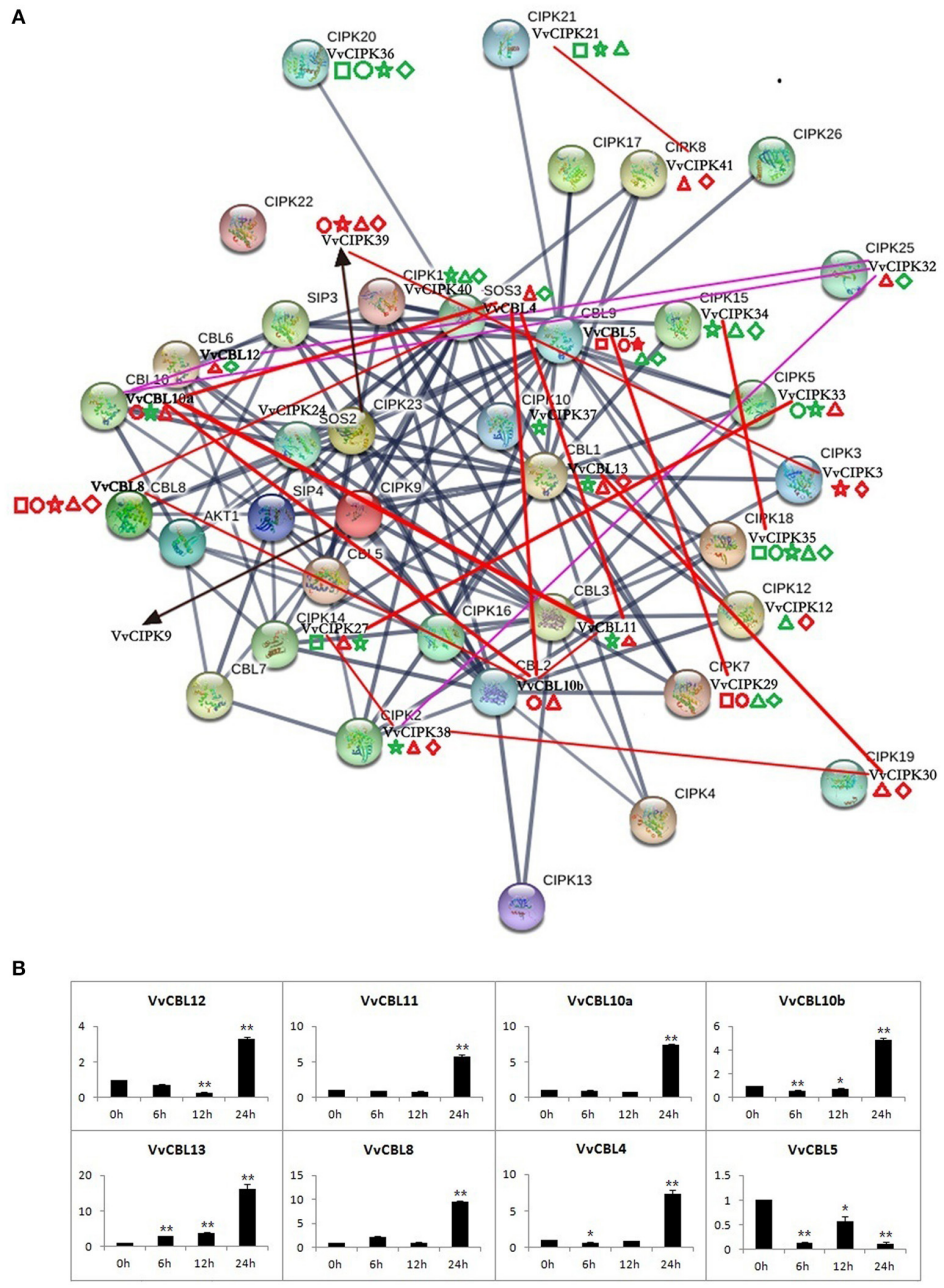
The protein interaction analysis of CBLs and CIPKs in Arabidopsis and grapevine **(A)**, as well as expression profile of *VvCBLs* in response to low temperature stress **(B)**. The dark blue lines represent interaction of AtCBL and AtCIPK proteins. Purple and red lines represent the co-expression relationships between VvCBL and VvCIPK genes based on the microarray data and qRT-PCR data, respectively. The thicker lines denote the higher confidence. The square, circle, pentagram, triangle, and rhombus indicate the gene expression patterns in responding to salt, PEG, low potassium, cold, and heat stress, respectively. The red shape is three-fold changes up-regulated and green is down-regulated.

Previous studies have demonstrated the important roles that *CBL* and *CIPK* genes play in plant response to abiotic stresses. Therefore, qRT-PCR analysis was conducted to characterize the expression profiles of grapevine *CBL* and *CIPK* genes when plants were subjected to various stress conditions. Transcript levels of all *VvCBL* and *VvCIPK* were characterized in 6-week-old grapevine leaves from plants that were subjected to various stress treatments, including ion stress including salt (NaCl; 200 mM) and low potassium (LK; 100 μM), osmotic stress (PEG; 10%), and low (4°C) and high (42°C) temperature stresses. As shown in Figure 5A and Figure S4, transcript levels of all *VvCBL* and *VvCIPK* genes were altered in response to the various stress treatments, and Figure 6A also show the more than three-fold changes up/down regulated genes. *VvCBL10a* and *VvCBL10b* of subgroup I were derived from tandem duplication, they were up-regulated by salt, PEG and cold stress, and were down-regulated by heat stress, however, *VvCBL10a* was down-regulated and *VvCBL10b* was up-regulated by LK stress (Figures 2–4). *VvCBL11* and *VvCBL12* of subgroup II have same expression profile, they were up-regulated by salt, PEG and cold stress, and were down-regulated by LK and heat stress (Figures 2, 4). *VvCBL5* and *VvCBL8* of subgroup IV were derived from tandem duplication, they were up-regulated by salt, PEG and LK stress, but *VvCBL5* was down-regulated and *VvCBL8* was up-regulated by temperature stress (Figures 2–4). Besides, *VvCBL8* was highly induced in response to most of the stress treatments (Figure 4).

*VvCIPK24, VvCIPK39*, and *VvCIPK3* were up-regulated by almost all stress treatments which were intron-rich clade of subgroup A (Figures 2, 4). In contrast, *VvCIPK34, VvCIPK35*, and *VvCIPK36* of subgroup C were down-regulated in response to almost all of the stress treatments (Figures 2–4). *VvCIPK12* which was derived from tandem duplication with *VvCIPK36*, was highly up-regulated by high temperature stress (Figures 3, 4). *VvCIPK27* which was derived from tandem duplication with *VvCIPK34*, were down-regulated by salt, PEG, and LK stress but up-regulated by cold and heat stress (Figures 3, 4). The expression profile of *VvCIPK28* is same to *VvCIPK27* expect the cold stress, as was derived from segmental duplication with *VvCIPK27* (Figures 3, 4). *VvCIPK38* which was derived from tandem duplication with *VvCIPK28*, was highly up-regulated in response to temperature stress (Figures 3, 4). But *VvCIPK37* which was derived from segmental duplication with *VvCIPK38*, was down-regulated by temperature, especially heat stress (Figures 3, 4). Besides, *VvCIPK33* and *VvCIPK30*, which were derived from tandem duplication, have same expression profile, were down-regulated by salt, PEG and LK stress and up-regulated by temperature stress (Figures 3, 4).

Studies have also reported the participation of *CBL* and *CIPK* genes in different development processes (Tripathi et al., 2009; Kanwar et al., 2014). Therefore, the expression pattern of *CBL* and *CIPK* genes in different grapevine tissues and organs was examined in order to obtain more insight into their role in plant growth and development. Figure 5B presents a heat map of the expression pattern of *VvCBL* and *VvCIPK* genes based on microarray data from 54 grapevine samples (Fasoli et al., 2012). Specific information about the different samples is provided in Table S6. The genes were ordered based on a hierarchical clustering analysis. As illustrated in Figure 5B, *VvCIPK39, VvCIPK37*, and *VvCIPK3* have a high level of expression throughout the various developmental stages of different grapevine organs and tissues. In contrast, *VvCBL8, VvCIPK35, VvCBL5* exhibited low levels of expression in the microarray data. These data collectively illustrate that grapevine *CBL* and *CIPK* respond to developmental stimuli.

### The divergent protein interactions of CBL-CIPKs between grapevine and arabidopsis

Figure 6A illustrates the networks of high confidence of interactive Arabidopsis CBL and CIPK proteins using STRING software, and identifies their VvCBL and VvCIPK homolog proteins by reciprocal best BLASTP analysis. The expression profiles of *VvCBL* and *VvCIPK* in response to abiotic stresses and the gene pairs with PCC value ≥0.7 were also intuitively added to the figure. The different interaction networks of CBL and CIPK proteins suggest that divergent mechanisms involving specific CBL/CIPK proteins may be present in Arabidopsis and grape.

The Pearson's correlation coefficient (PCC) among *VvCBL* and *VvCIPK* genes was calculated based on the qRT-PCR data in order to further characterize the co-expression relationship between *VvCBLs* and *VvCIPKs*. As shown in Table S7, the PCC value of 20 gene pairs was more than 0.7 at the 0.01 level (2-tailed), and all these pairs exhibited a significant positive correlation. Importantly, the *VvCBL13* and *VvCIPK30* pair exhibited both a strong PCC value and a similar expression response in the qRT-PCR data as both were significantly up-regulated in response to both low and high temperature stress (Figure 6A, Table S7). *VvCBL5* and *VvCIPK29* pair also had both a strong PCC value and a similar expression pattern in the qRT-PCR data. They were both significantly up-regulated in response to ion (salt and low K^+^) and osmotic (PEG) stress and significantly down-regulated during some of the time points of temperature stress (Figure 6A, Table S7). Besides, as shown in Figure 6A, 11 strong correlated gene pairs (55%) were up-regulated by low temperature stress, including *VvCBL4/VvCBL8, VvCBL10b/VvCBL8, VvCBL10b/VvCBL11, VvCBL13/VvCIPK30, VvCBL10a/VvCBL10b, VvCBL4/VvCBL11, VvCBL10a/VvCBL4, VvCBL10b/VvCBL4, VvCBL10a/VvCBL11*. Most of them were *VvCBL* genes, so the expression profile of *VvCBLs* in response to low temperature were more intuitively drafted as Figure 6B individually, we found that all *VvCBLs* were highly up-regulated by cold stress at 24 h except *VvCBL5*.

PCC and co-expression network analysis were also carried out using the *VvCBL* and *VvCIPK* microarray data. Results indicated that 56 pairs of 28 *VvCBLs* and *VvCIPKs* exhibited a significant correlation. The PCC values of 42 pairs were ≥0.35 and exhibited a significant positive correlation and the PCC values of 14 pairs were ≤ -0.35 and exhibited a significant negative correlation. Among the significant correlations, the PCC values of four gene pairs were ≥0.7 and those of four gene pairs were ≤ -0.7. PCC of *VvCIPK32/VvCIPK38, VvCBL12/VvCIPK32, VvCBL10a/VvCIPK32*, and *VvCBL12/VvCBL10a* exhibited a positive correlation each with a PCC ≥0.7.

## Discussion

Calcium plays a major role in regulating signal transduction pathways that are activated in response to various environmental stimuli and development processes. Calcium sensors, such as CBLs, work together with their target kinases, CIPKs, to regulate plant development phases and the response to environmental stress (Kudla et al., 2010). Much of the research into the function of CBL and CIPK families has been analyzed in model plants and major crops, such as Arabidopsis, rice, poplar, and other species (Qiu et al., 2002; Zhang et al., 2008; Kanwar et al., 2014), but research in grapevine is limited.

In the present study, 8 *CBL* and 20 *CIPK* genes were identified in the grapevine genome using a comprehensive means (see Materials and Methods). Multiple sequence alignment of VvCBLs showed that all CBLs in grapevine have four EF hands and the link spaces between EF hands are highly conserved through all VvCBLs, in which, there are 22 amino acids between EF1 and EF2, 25 amino acids between EF2 and EF3, and 32 amino acids between EF3 and EF4. Our results are consistent with structural feature of CBLs in Arabidopsis and other species (Kolukisaoglu et al., 2004; Lyzenga et al., 2013; Yu et al., 2014; Mohanta et al., 2015). Furthermore, the recent characterized motif PFPF, which is important for the phosphorylation cascades of CBLs (Ames et al., 1997; Kanwar et al., 2014), was also identified in VvCBLs, and found to be conserved through all the members of grapevine CBL gene family. Similar to OsCBLs, all VvCBLs possess conserved serine residue in the PFPF motif (Kanwar et al., 2014). Besides, three VvCBLs, including VvCBL13, VvCBL4, and VvCBL5, were identified to have a myristoylation site in the N-terminal sequence, which is required to exhibit the calcium-dependent membrane association of recover in proteins by switching calcium with myristoyl (Du et al., 2011).

Previous research has shown that CIPK proteins include a conserved N-terminal catalytic kinase domain and a C-terminal regulatory domain (Kim et al., 2000). The N-terminal catalytic kinase domain has an ATP binding site and an activation loop. The C-terminal regulatory domain contains a NAF/FISL motif that mediates the interaction between CIPKs and CBLs, as well as interaction between CIPKs and type 2C protein phosphates (PP2C) through the PPI motif (Ohta et al., 2003). In our study, all VvCIPKs possess an activation domain in the N-terminal sequence and a NAF domain in the C-terminal sequence, except for VvCIPK35, which seems to be missing both the C-terminal regulatory domain and the NAF domain. The phylogenetic analysis of VvCIPK35, however, does indicate to have high homology with other Arabidopsis and grapevine CIPKs (Table S1, Figure S1B). Therefore, VvCIPK35 was still considered to represent a valid VvCIPK.

Based on the phylogenetic analysis, grapevine CBLs and CIPKs were divided into four and five subgroups, respectively (Figure 1), along with CBLs and CIPKs in Arabidopsis and poplar (Kolukisaoglu et al., 2004). Importantly, some closely-related orthologous pairs of CBLs and CIPKs between grapevine and Arabidopsis were identified. Such as VvCBL8/AtCBL8, VvCBL10a, -10b/ATCBL10, VvCBL4/AtCBL4, VvCBL5/AtCBL5, VvCIPK3/AtCIPK3, VvCIPK9/AtCIPK9, VvCIPK12/AtCIPK12, VvCIPK21/AtCIPK21, VvCIPK24/AtCIPK24 (Figure 1). These results suggest that closely-related orthologous genes in grapevine may have conserved function among different species. On the contrary, most VvCIPKs and CBLs were not found to be confident orthologous pairs between grapevine and Arabidopsis, indicating that VvCBLs and VvCIPKs might functionally diverged from Arabidopsis or even other species (Figure 1).

Since gene structure like intron/exon organizations and intron types are typical imprints of the evolution with in some gene families (Boudet et al., 2001; Wang et al., 2013; Liu et al., 2014). Certain degrees of similarity in gene structure were observed within each subgroup in our present study. Interestingly, most members of subgroup B, C, D, and E of *VvCIPKs* are intron less or intron poor comparing to subgroup A (Figure 2D), and VvCIPK gene family was thereby divided into intron poor clade (including subgroup B, C, D, and E) and intron rich clade (subgroup A), respectively. This feature of gene structure in *CIPK* genes was also conserved in Arabidopsis, rice, maize, poplar, and soybean (Kolukisaoglu et al., 2004; Chen et al., 2011; Ye et al., 2013; Zhu et al., 2016). These data indicate that intron gain and loss events have played an important role in the evolution of the CIPK family.

Duplication and divergence play an important role in expansion and evolution of gene families (Hughes, 1994; Vision et al., 2000). The gene duplication events between grapevine *CBL* and *CIPK* genes in grapevine genome were surveyed to gain more insight into their evolutionary course. Our results indicated that 4 (50%) out of 8 VvCBL genes and 8 (40%) out of 20 *VvCIPK* genes were originated from tandem duplication events, and 5 (25%) out of 20 *VvCIPK* genes were derived from segmental duplication events, suggesting that gene duplication has been the mainly evolutionary force underlying the expansion of the *CBL* and *CIPK* gene families in grapevine (Figure 3). Interestingly, the duplication events in *VvCIPK* gene family only exist in intron-poor clade, suggesting that intron-poor clade of *VvCIPK* gene family may play more specific role to fulfill the species characteristics of grapevine. Furthermore, the Ka/Ks ratio of *VvCBL* and *VvCIPK* genes shown that duplicated *VvCBL* genes were driven by positive selection as Ka/Ks ratio >1, whereas duplicated *VvCIPK* genes were driven by purifying selection as Ka/Ks ratio <1 (Lynch and Conery, 2000), suggesting that the duplication events have accelerated the evolution of *VvCBL* genes (Table S2). However, *VvCIPK* genes were substitutional eliminated and selection was limited by natural selection during their evolution course. Besides, all duplication events of these *VvCBL* and *VvCIPK* genes were occurred in about 8 to 17 Mya (Figure 4, Table S2).

We also detected 2, 4, and 7 *CIPK* orthologous gene pairs between grapevine and Arabidopsis, grapevine and rice, grapevine and poplar, respectively (Figure 4, Table S3). However, no *CBL* orthologous gene pairs between grapevine and other species examined. It suggested that *CBL* were much more conserved than CIPK during the evolution. Moreover, the duplicated *CIPK* genes among these species were under strong purifying selection as Ka/Ks ratio <1 (Lynch and Conery, 2000). In addition, the average Ks value of *CIPK* gene pairs between grapevine and Arabidopsis, rice and poplar were 1.89, 2.31, and 1.37 (Table S3), respectively, suggested that more recent divergence occurred between grapevine and poplar, followed by Arabidopsis and further relationship between grapevine and rice. This is consistent with the analysis by phylogeny (Figure 1).

Previous studies have demonstrated that the CBL, CIPK, or CBL-CIPK complex functions in regulating the development phases of plant growth and the response to environmental stress (Kanwar et al., 2014; Yu et al., 2014). For example, AtCBL10 was reported to interact with AtCIPK24 in response to salt stress (Kim et al., 2007) and was shown to compete with AtCIPK23 for binding to AKT1, thus negatively modulating the activity of AKT (Ren et al., 2013). VvCBL10a and VvCBL10b, which were orthologous genes of AtCBL10, were up-regulated by salt, PEG and cold stress, and down-regulated by heat stress, but VvCBL10a was down-regulated and VvCBL10b was up-regulated by LK stress (Figure 5, Figure S4). Overexpression of AtCBL5 confers osmotic or drought stress tolerance (Cheong et al., 2010). The ortholog gene VvCBL5 was up-regulated by salt, PEG and LK stress, and drought related element MBS was found in the promoter region of VvCBL5 (Figure 5, Figure S4, Table S4). AtCBL8 was reported to interact with AtCIPK23, activated HAK5, and increase the affinity of K+ (Ragel et al., 2015). The orthologous gene VvCBL8 was up-regulated by almost all stress conditions (Figure 5, Figure S4). AtCBL4 (AtSOS3) typically interacts with AtCIPK24 (AtSOS2) and activates an Na+/H+ antiporter (AtSOS1) antiporter and an H+-ATPase, resulting in enhanced salt tolerance (Qiu et al., 2002). VvCBL4 and VvCIPK24, which were orthologous genes of AtCBL4 and AtCIPK24, were up-regulated by salt, PEG, LK and cold stress, drought related element MBS was also found in the promoter region of VvCBL4 (Figure 5, Figure S4, Table S4). AtCIPK3 was reported to regulates ABA and cold signal transduction (Kim et al., 2003). The orthologous gene VvCIPK3 was up-regulated by almost all stress conditions, drought related element MBS and salicylic acid response TCA-element were also found in the VvCIPK3 promoter region (Figure 5, Figure S4, Table S4). AtCIPK21 was reported regulates osmotic and salt stress responses (Pandey et al., 2015). However, the orthologous gene VvCIPK21 was down-regulated by salt, PEG, LK, cold stress, and was up-regulated by heat stress. In addition to CBL-CIPK complexes, however, Arabidopsis CBL3 also interacts with 5′-methylthioadenosine nucleosidase in a calcium-dependent manner (Oh et al., 2008). AtCBL10 functions independently of the SOS and AKT pathway (Ren et al., 2013; Monihan et al., 2016). This indicates that CBLs can combine and affect downstream components independent of CIPK proteins. Besides, based on the analysis of microarray data, many grapevine CBL and CIPK genes exhibit a high level of expression in various grape organs and tissues across different developmental stages (Figure 5B). However, little evidence of a strong correlation between the expression of CBL and CIPK gene pairs was evident (Figure 6A, Table S7). In contrast, the expression of VvCBLs and VvCIPKs in response to high salt, drought, low potassium, and low and high temperature stress as determined by qRT-PCR was much more diversified (Figure 5A). A strong correlation in the expression (>seven-fold) of gene pairs was also evident (Figure 6A, Table S7). Therefore, it may be speculated that grapevine CBL-CIPK complexes may be more responsive in regulating the response to abiotic stimuli than in regulating plant development.

In the present study, several grapevine CBL and CIPK genes were found to be specifically up-regulated by temperature stress. This included VvCIPK38, VvCIPK30, and VvCBL13 (Figure 5, Figure S4). Notably, the VvCBL13/VvCIPK30 gene pair had a significantly strong PCC value (Table S7). VvCIPK29 and VvCBL5 were specifically up-regulated by salt, PEG and LK stress and also had a strong PCC value. VvCBL13/VvCIPK30 and VvCBL5/VvCIPK29 complexes may play a role in response to abiotic stress, but this result needs further verification. VvCIPK35 was down-regulated in all of the developmental stages and all examined abiotic stress conditions (Figure 5, Figure S4). Lastly, strong PCC values for VvCBL/VvCIPK gene pairs were used to draw a co-expression network based on the interaction network of Arabidopsis CBL and CIPK genes (Figure 6A, Table S7). Our results suggest that the divergent protein interactions of CBL-CIPKs might exist between grapevine and Arabidopsis in their response to abiotic stresses. In addition, the expression pattern of VvCBLs showed that all VvCBLs were up-regulated by low temperature stress at 24 h treatment except VvCBL5 (Figure 6B), suggested the important roles of VvCBLs in regulation of pant tolerance to low temperature stress.

## Author contributions

JL, YX designed the experiment, YX with help of CD performed the experiment, processed the data, and wrote the manuscript, CD, JL, and ZC revised the manuscript.

### Conflict of interest statement

The authors declare that the research was conducted in the absence of any commercial or financial relationships that could be construed as a potential conflict of interest.
