# Supplementary figures and images for: The CBL and CIPK Gene Family in Grapevine (Vitis vinifera): Genome-Wide Analysis and Expression Profiles in Response to Various Abiotic Stresses
Source: Front Plant Sci. 2017 Jun 9;8:978. doi: 10.3389/fpls.2017.00978 (PMC5465270; doi:10.3389/fpls.2017.00978)

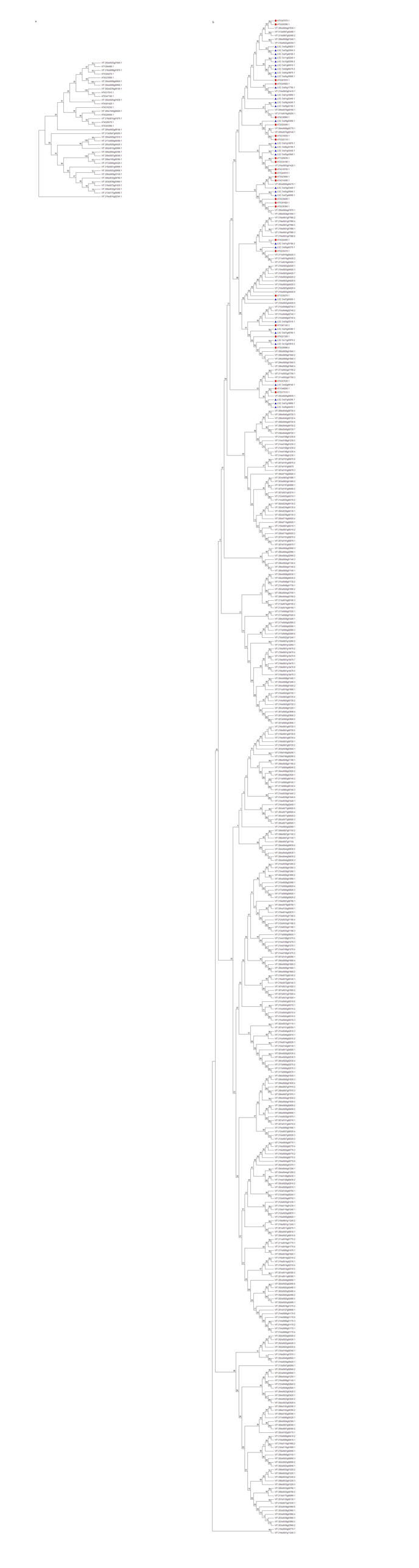

Supplement: Supplementary file 8 [file Image1.JPEG]

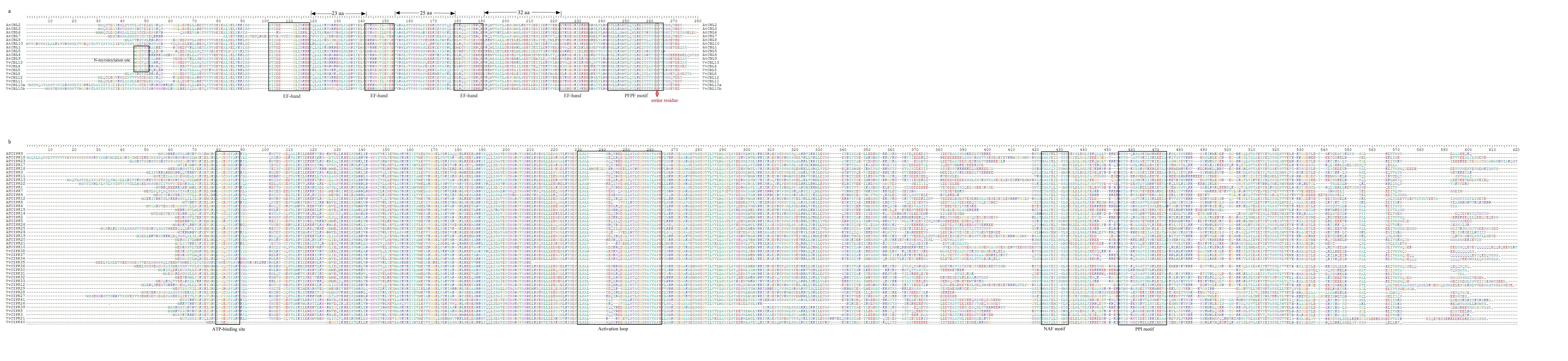

Supplement: Supplementary file 9 [file Image2.JPEG]

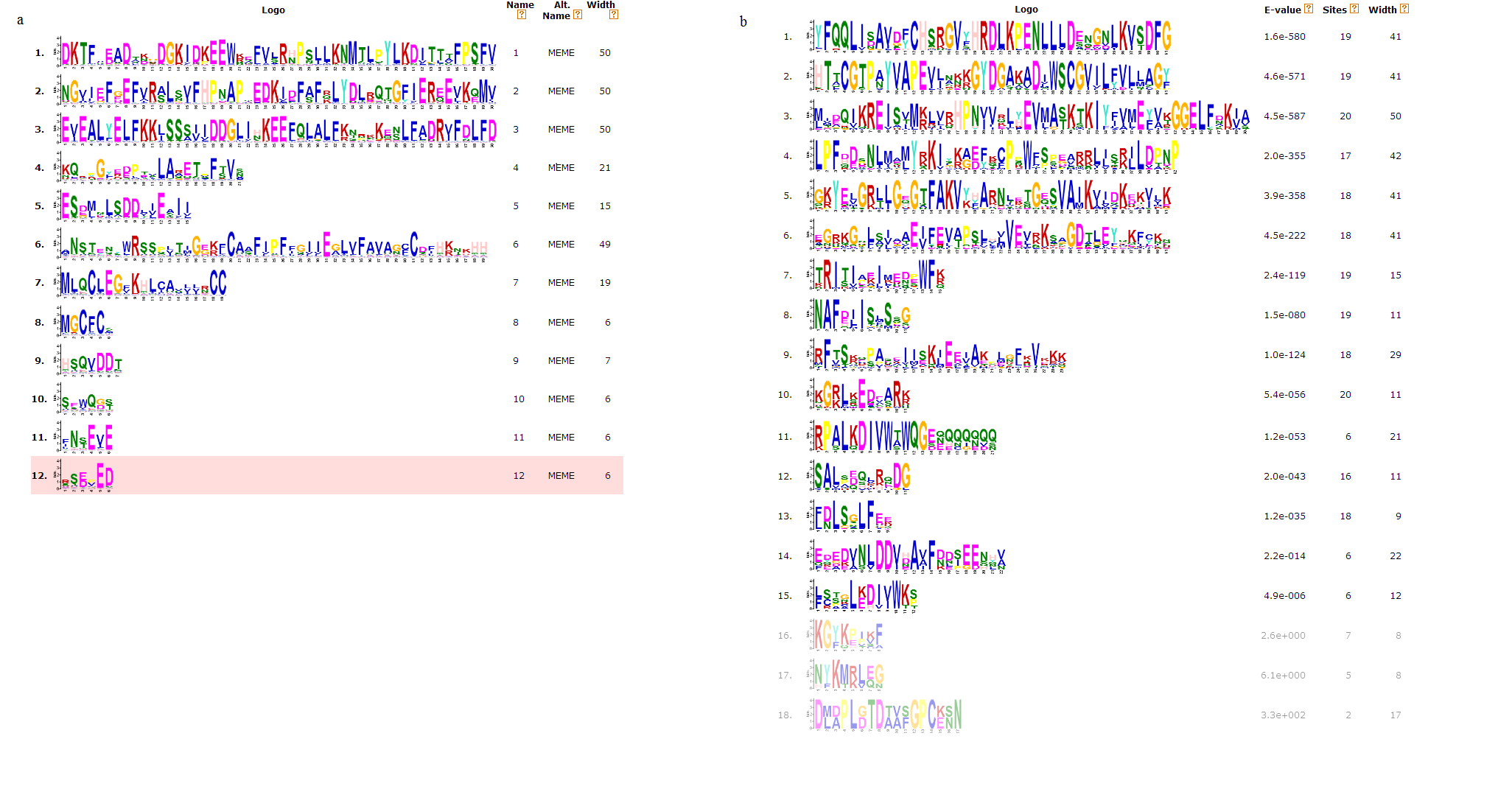

Supplement: Supplementary file 10 [file Image3.PNG]

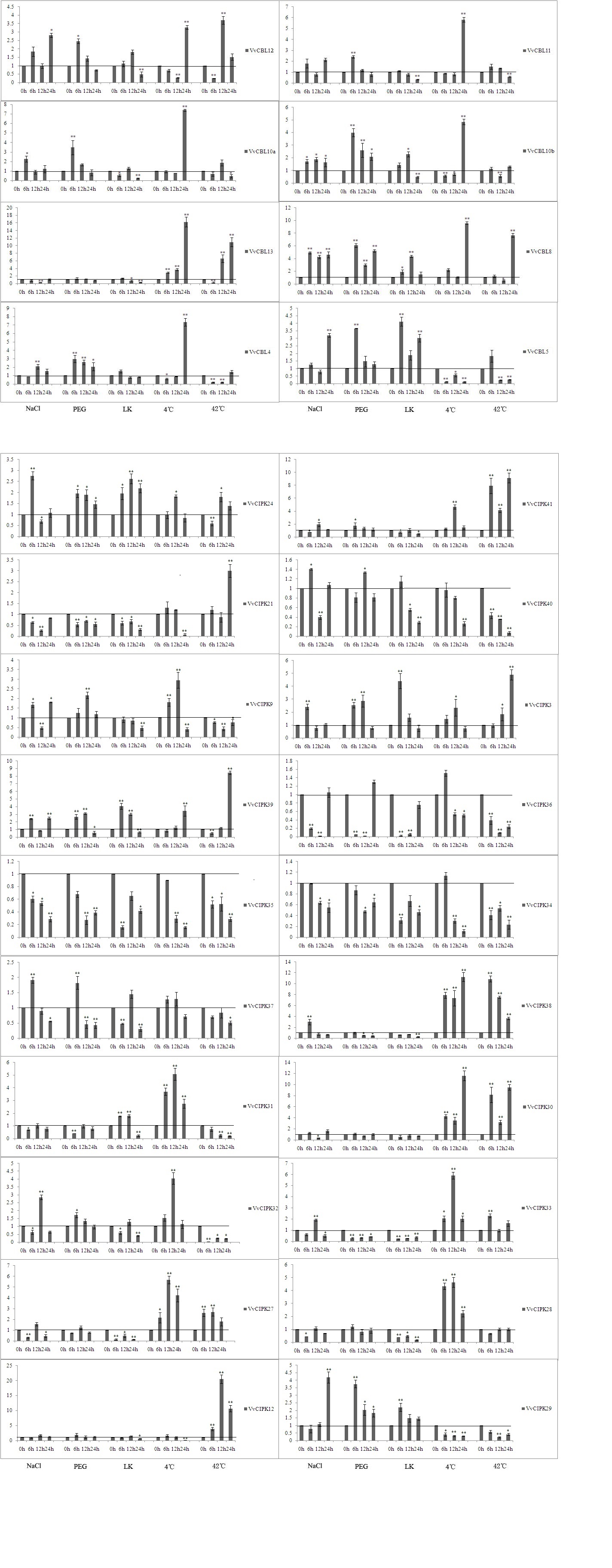

Supplement: Supplementary file 11 [file Image4.JPEG]
